# Effect of Gestational Weight Gain on Associations Between Maternal Thyroid Hormones and Birth Outcomes

**DOI:** 10.3389/fendo.2020.00610

**Published:** 2020-09-03

**Authors:** Bin Zhou, Yao Chen, Wen-Qian Cai, Ling Liu, Xi-Jiang Hu

**Affiliations:** ^1^Eugenic Genetics Laboratory, Wuhan Children's Hospital (Wuhan Maternal and Child Healthcare Hospital), Tongji Medical College, Huazhong University of Science & Technology, Wuhan, China; ^2^Technology Department, Wuhan Pengxiang Medical Equipment Co., Ltd., Wuhan, China

**Keywords:** thyroid function, birth weight, low birth weight, gestational weight gain, birth length

## Abstract

**Purpose:** The aim was to investigate the associations between maternal thyroid parameters within the normal ranges during early pregnancy and birth outcomes, and further to examine whether the associations were modified by gestational weight gain (GWG).

**Methods:** Maternal serum thyroid-stimulating hormone (TSH), free thyroxine (FT4), and free triiodothyronine (FT3) concentrations within the normal ranges during early pregnancy were measured from 8,107 pregnant women in Wuhan, China. The associations between maternal thyroid parameters and birth outcomes (birth weight, birth length, and low birth weight) were analyzed using multivariable adjusted regression models, and effect modification by pre-pregnancy body mass index (BMI) category and GWG were further evaluated.

**Results:** Maternal TSH and FT4 concentrations were negatively associated with birth weight, and the latter only occurred in normal weigh women with inadequate and excessive GWG, as well as in both underweight and overweight women with excessive GWG (e.g., β = −359.33 g, 95% CI: −700.95, −17.72 in underweight women with excessive GWG for per unit increase of FT4 concentrations). Moreover, maternal FT4 and FT3 concentrations were associated with increased risk for low birth weight, and the latter only occurred in normal weigh women with inadequate GWG (OR = 2.52, 95% CI: 1.00, 6.36 for per unit increase of FT3 concentrations). These associations still persist when maternal thyroid parameters were modeled as quintiles.

**Main conclusion:** Maternal normal thyroid function during early pregnancy with excessive and inadequate GWG may adversely influence fetal growth.

## Introduction

Maternal thyroid function plays a vital role in ensuring placentation and regulating fetal organ development and maturation (1–3). Specially, the fetus cannot synthesis thyroid hormones and completely depends on maternal supply in the first trimester (4, 5). To meet the demand of developing fetus, temporal peak of human chorionic gondotropin (hCG) promotes the synthesis of maternal thyroid hormones at the early of gestation, resulting in increased production in free triiodothyronine (FT3), free thyroxine (FT4), and thyroid-stimulating hormone (TSH). Alternations in maternal thyroid hormones during pregnancy, particularly at the early of gestation, is expected to influence the developmental and growth of a fetus.

Accumulating evidence has consistently demonstrated that abnormal maternal thyroid function such as overt hypothyroidism and hyperthyroidism during pregnancy are related with increased risk of adverse obstetrical and offspring outcomes including preeclampsia, gestational diabetes, placental abruption, preterm birth, fetal death, and low birth weight (6–14). Several studies have also evaluated the effects of subclinical maternal thyroid dysfunctions on fetal outcomes including spontaneous abortion, preterm birth, low birth weight, low head circumference, intrauterine growth retardation, and fetal death (15–18), with inconsistent results. However, it remains poorly understood for the effects of maternal thyroid hormones within the normal ranges during pregnancy on fetal growth (19–22). Furthermore, previous studies primarily focus on TSH and FT4, whereas the data regarding the effect of FT3, as the principal bioactive thyroid hormone, on fetal growth is lacking (22).

Apart from maternal thyroid function, gestational weight gain (GWG) has also been shown to affect fetal growth (23). The Institute of Medicine (IOM) recommends the GWG ranges based on pre-pregnancy body mass index (BMI) category to reduce the incidence of adverse birth outcomes (24). Considerable studies have shown that inadequate and excessive GWG are related with increased risk of low birth weight, preterm birth, and overweight (23, 25, 26). However, to the best of our knowledge, no studies to date have examined whether the GWG modifies the effects of maternal thyroid hormones during pregnancy on birth outcomes.

In the present study, we investigated the associations between maternal serum thyroid parameters within the normal ranges during early pregnancy and birth outcomes in a larger population-based prospective study in Wuhan, China. Furthermore, we examined whether the associations were modified by GWG.

## Subjects and Methods

### Study Subjects

Pregnant women during the first prenatal visit [median 13.6 weeks of gestation, standard deviation (SD) = 1.8 weeks] were invited to participate in this study in the Wuhan Children's Hospital (Wuhan Maternal and Child Healthcare Hospital) between November 4, 2014 and April 17, 2018. A total of 12,544 pregnant women during the study period completed the questionnaires containing the information about demographics, health status and obstetric history. We excluded 2,863 pregnant women who had chronic diseases, thyroid related diseases and pregnancy complications, as well as 415 pregnant women who gave birth to birth defects perinatal death and twins. Additionally, 29 pregnant women reported that they were smokers or alcohol consumers during the pregnancy were further excluded, resulting in 9,237 study subjects in this study. Among them, 8,107 pregnant women who had the serum thyroid parameters within the normal ranges were retained in the final analysis. The authors have obtained both informed consent and ethics committee approval of Wuhan Children's Hospital (Wuhan Maternal and Child Healthcare Hospital) for studies on patients, patient records, or volunteers.

### Thyroid Hormone Measurements

Five milliliter of blood samples from pregnant women in their first prenatal examination in the hospital were collected and then were centrifuged to obtain serum. Maternal serum thyroid hormones including TSH, FT3, and FT4 were measured within 24 h by DxI800 Immunoassay System (Beckman CoulterUniCel). The inter-assay coefficients of variation (CVs) at low and high concentrations ranged from 1.88 to 3.92% for FT3, 1.66–2.58% for FT4, and 2.43–2.80% for TSH. The between-assay CVs were 1.60–2.85% for FT3, 1.70–3.30% for FT4, and 2.50–3.80% for TSH. A total of 8,107 pregnant women who had their serum thyroid hormone concentrations within the normal ranges according to the percentile distribution of serum thyroid hormones (TSH, FT3, and FT4 between the 2.5th and 97.5th percentiles) among the whole population. The 2.5th−97.5th percentiles ranges of FT3, FT4 and TSH concentrations were 1.93–3.85 ng/L, 0.55–0.98 ng/dL, and 0.10–3.24 uIU/mL, respectively.

### Birth Outcomes

Birth outcomes including birth length (in centimeters), birth weight (in grams), gestational age (in weeks), and fetal sex were collected from the Wuhan delivery system and the child care system in the Wuhan Children's Hospital (Wuhan Maternal and Child Healthcare Hospital). Gestational age was calculated as the difference between the date of the last menstruation and the date of delivery. Low birth weight was defined as birth weight below 2,500 g.

### Covariates

Maternal characteristics including age, height, pre-pregnancy, and prenatal weight, mode of delivery, parity, education, and high risk pregnancy were obtained from the questionnaires or the Wuhan delivery system, and the child care system. Body mass index (BMI) was calculated as weight divided by squared height (kg/m^2^). The pregnant women were defined as high risk pregnancy if they meet one of the following criteria: pre-pregnancy weight <40 or>80 kg; height ≤ 1.45 m; age <18 or ≥35 years; malformations of the birth canal or narrow pelvis; abnormal uterine development; thoracic spine deformities; abnormal pregnancy history; pregnancy complication; abnormal conditions during the pregnancy such as threatened abortion, placental abruption, preeclampsia, dyspnea, and acute abdominal pain. GWG (in kilograms) was calculated as the difference between the prenatal weight, and the pre-pregnancy weight. According to the IOM guidelines of 2009 (24), GWG was classified into three categories (inadequate, adequate, and excessive) based on pre-pregnancy BMI (Table S1).

### Statistical Analysis

We used the Predictive Analytics Suite Workstation (PASW) version 18.0 for the data analysis. Descriptive statistics for demographics, birth outcomes, and maternal thyroid hormone concentrations were conducted. Statistical significance was defined as a *p* < 0.05.

Multivariable linear models were used to investigate the associations of continuous maternal thyroid hormones (TSH, FT3, and FT4) with birth weight, and birth length. We also categorized maternal thyroid hormones into quintiles to investigate the possible dose-response associations. A test of trend across quintiles of maternal thyroid hormones modeled as integer values (0–4) was examined and the first quintile was considered as the reference. Logistic regression models were applied to estimate odds ratio (ORs) and 95% confidence intervals (CIs) for the associations between maternal thyroid hormones and risk for low birth weight. The above associations stratified by pre-pregnancy BMI category and GWG were further conducted. The cross-product term between maternal thyroid hormones and GWG was used to assess the interactions. Because the number of obese women (*n* = 114) in our study population was limited, we did not include them into the stratification analysis. We also only conducted the stratification analysis for low birth weight among normal weight women due to the limited cases.

Covariates were included in the final regression models based on statistical and biological considerations. Those covariates were entered into the final multivariable adjusted models with *p* < 0.15 in univariate associations with birth outcomes. Finally, all the regression models were adjusted for the following variables: maternal height, GWG and pre-pregnancy weight (continuous), maternal age (<25, 25~ <35, >35 years), maternal education (high school and below, college and above), mode of delivery (natural childbirth, cesarean section), parity (no child, ≥ one child), high risk pregnancy (yes, no), infant's sex (male, female) and gestational age (<37, ≥37 weeks). We did not adjust both of GWG and pre-pregnancy weight in the stratification analysis.

## Results

### Characteristics of the Study Population

The characteristics of mothers and newborns are described in Table 1. The mean (SD) maternal age and pre-pregnancy BMI were 29.0 (3.8) years and 21.0 (2.9) kg/m^2^, respectively. Of the 8,107 women, 1,358 (16.8%) were categorized as underweight, 6,010 (74.1%) as normal weight, 625 (7.7%) as overweight and 114 (1.4%) as obese. More than half of the women were nulliparous (71.2%) and well-educated (65.4%). The mean (SD) birth length, birth weight and gestational age were 50.2 (1.6) cm, 3,327 (413) g, and 39.0 weeks (1.3), respectively. A total of 178 newborns (2.2%) were low birth weight. The mean (SD) maternal TSH, FT4, and FT3 concentrations within the normal ranges during early pregnancy were 1.35 (0.71) uIU/mL, 0.76 (0.09) ng/dL, and 3.00 (0.37) ng/L, respectively.

**Table 1:** Characteristics of study population (*N* = 8,107^a^).

**Characteristic**	**Mean (SD) or *n* (%)**
Maternal age, years	29.0 (3.8)
Maternal height, cm	160.8 (5.0)
Gestational weight gain (GWG), kg	15.3 (5.9)
Pre-pregnancy body mass index (BMI), kg/m^2^	21.0 (2.9)
Underweight	1,358 (16.8)
Normal weight	6,010 (74.1)
Overweight	625 (7.7)
Obese	114 (1.4)
Mode of delivery	
Natural childbirth	4,038 (49.8%)
Cesarean section	4,029 (49.7%)
Maternal education	
High school and below	2,809 (34.6%)
College and above	5,298 (65.4%)
Parity	
0	5,770 (71.2%)
≥1	2,337 (28.8%)
Infant sex	
Male	4,248 (52.4%)
Female	3,859 (47.6%)
Gestational age, weeks	39.0 (1.3)
<37	268 (3.3%)
≥37	7,839 (96.7%)
High risk pregnancy	
Yes	4,277 (52.8%)
No	3,830 (47.2%)
Maternal TSH, uIU/mL	1.35 (0.71)
Maternal FT4, ng/dL	0.76 (0.09)
Maternal FT3, ng/L	3.00 (0.37)
Birth weight, g	3,327 (413)
Birth height, cm	50.2 (1.6)
Low birth weight	178 (2.2%)

a *One missing pre-pregnancy BMI, 4 missing GWG, and 40 missing mode of delivery in this study population*.

### Associations Between Maternal Thyroid Hormones and Birth Outcomes

Multivariable-adjusted associations between maternal thyroid hormones and birth outcomes are summarized in Table 2. We found that maternal TSH and FT4 concentrations were negatively associated with birth weight. The birth weight decreased by 13.49 g (95% CI: −24.71, −2.27) and by 189.88 g (95% CI, −275.28, −104.48) for every unit increase in TSH and FT4 concentrations, respectively. Significant dose-dependent relationships were still found across increasing quintiles of maternal TSH and FT4 concentrations with decreasing birth weight (both p for trends < 0.05). We did not observe significant associations between maternal FT3 concentrations and birth weight.

**Table 2:** Associations between maternal thyroid parameters during early pregnancy and birth outcomes ^a^(*N* = 8,107).

**Variables**	**Birth weight**	**Birth length**	**Low birth weight**
	**β (95% CI)**	**β (95% CI)**	**OR (95% CI)**
TSH continuous	**−13.49 (−24.71**, **−2.27)**	−0.02 (−0.07, −0.02)	0.96 (0.75, 1.22)
TSH quintiles			
Q1	Reference	Reference	Reference
Q2	−12.10 (−37.22, 13.03)	−0.01 (−0.09, 0.07)	0.819 (0.48, 1.40)
Q3	−14.10 (−39.25, 11.05)	−0.04 (−0.12, 0.04)	0.84 (0.50, 1.42)
Q4	−20.95 (−46.17, 4.28)	0.03 (−0.06, 0.11)	0.88 (0.51, 1.51)
Q5	**−29.84 (−55.06**, **−4.62)**	−0.03 (−0.11, 0.05)	0.80 (0.46, 1.38)
*P* for trend	**0.02**	0.34	0.53
FT4 continuous	**−189.88 (−275.28**, **−104.48)**	**−0.50 (−0.85**, **−0.15)**	**8.98 (1.39, 57.88)**
FT4 quintiles			
Q1	Reference	Reference	Reference
Q2	−0.40 (−24.98, 24.17)	0.06 (−0.02, 0.13)	1.50 (0.82, 2.75)
Q3	−19.26 (−44.514, 6.003)	0.03 (−0.049, 0.114)	1.65 (0.89, 3.07)
Q4	−25.21 (−50.74, 0.32)	−0.04 (−0.12, 0.04)	**2.13 (1.17, 3.89)**
Q5	**−48.09 (−73.69**, **−22.49)**	**−0.10 (−0.18**, **−0.02)**	**1.94 (1.06, 3.56)**
*P* for trend	** <0.001**	**0.01**	**0.02**
FT3 continuous	−1.96 (−23.96, 20.05)	−0.003 (−0.09, 0.09)	**1.97 (1.26, 3.09)**
FT3 quintiles			
Q1	Reference	Reference	Reference
Q2	−19.93 (−45.06, 5.21)	0.004 (−0.08, 0.08)	1.23 (0.68, 2.24)
Q3	−6.46 (−31.47, 18.56)	0.01 (−0.07, 0.09)	1.65 (0.92, 2.95)
Q4	−3.66 (−29.38, 22.06)	0.01 (−0.08, 0.09)	1.64 (0.90, 2.98)
Q5	−10.87 (−36.41, 14.69)	−0.01 (−0.10, 0.07)	**2.42 (1.37, 4.26)**
*P* for trend	0.84	0.94	**0.001**

a *Adjusted for maternal education, maternal height, maternal age, gestational age, GWG, pre-pregnancy BMI, parity, infant's sex, high risk pregnancy, and mode of delivery. Bold values are statistically significant*.

We observed that maternal FT4 and FT3 concentrations were associated with increased risk for low birth weight; the ORs were 8.98 (95% CI: 1.39, 57.88) and 1.97 (95% CI: 1.26, 3.09) for every unit increase in FT4 and FT3 concentrations, respectively. Significant dose-dependent relationships across increasing quintiles of maternal FT4 and FT3 concentrations with increasing risk for low birth weight were still found (both p for trends < 0.05). We found that maternal TSH concentrations were not significantly associated with increased risk for low birth weight.

We observed that maternal TSH and FT3 concentrations were not associated with birth length. However, we found that maternal FT4 concentrations were negatively associated with birth length. The birth length decreased by 0.50 cm (95% CI: −0.85, −0.15) for every unit increase in FT4 concentrations. Significant dose-dependent relationships were still observed across increasing quintiles of maternal FT4 concentrations with decreasing birth length (*p* for trend =0.01).

### Stratification Analysis by Pre-pregnancy BMI Category and GWG

Among normal weight women, inverse associations between maternal FT4 concentrations and birth weight were observed in excessive and inadequate GWG but not in adequate GWG, though no significant interactions were observed (Table 3). The birth weight decreased by 289.70 g (95% CI: −508.44, −70.96) and by 222.39 g (95% CI, −370.34, −74.45) for every unit increase in FT4 concentrations in excessive and inadequate GWG, respectively. Moreover, among normal weight women, maternal FT3 concentrations in relation to increased risk for low birth weight only observed in inadequate GWG but not in excessive and adequate GWG, though no significant interactions were observed; the OR was 2.52 (95% CI: 1.00, 6.36) for every unit increase in FT3 concentrations in inadequate GWG (Table 4). These associations still persisted when maternal FT3 concentrations were modeled as quintiles.

**Table 3:** Adjusted regression coefficients ^a^ for birth weight associated with maternal thyroid parameters during early pregnancy stratified by GWG among normal weight women (*N* = 6,010).

**Variables**	**GWG (kg)**			***P* for interaction**
	** <11.5 (*N* = 1,244) β (95% CI)**	**11.5**~**16.0 (*N* = 2,001) β (95% CI)**	**≥16.0 (*N* = 2,765) β (95% CI)**	
TSH continuous	−9.61 (−39.36, 20.15)	−14.38 (−36.47, 7.72)	−10.76 (−30.31, 8.79)	0.78
TSH quintiles				0.96
Q1	Reference	Reference	Reference	
Q2	0.30 (−63.35, 63.96)	6.67 (43.46, 56.80)	−3.31 (−47.86, 41.24)	
Q3	−34.15 (−99.30, 31.00)	3.97 (−45.71, 53.80)	−14.85 (−59.14, 29.45)	
Q4	−1.62 (−68.23, 64.98)	−9.31 (−59.64, 41.01)	−17.24 (−61.10, 26.61)	
Q5	−20.88 (−87.36, 45.60)	−19.37 (−69.64, 30.37)	−23.78 (−67.91, 20.35)	
*P* for trend	0.55	0.34	0.22	
FT4 continuous	**−289.70 (−508.44**, **−70.96)**	−130.24 (−300.48, 40.00)	**−222.39 (−370.34**, **−74.45)**	0.63
FT4 quintiles				0.45
Q1	Reference	Reference	Reference	
Q2	1.99 (−62.99, 66.98)	0.003 (−0.05, 0.05)	15.98 (−26.69, 58.65)	
Q3	−41.44 (−107.71, 24.84)	−0.01 (−0.06, 0.04)	1.45 (−42.05, 44.95)	
Q4	**−76.49 (−143.43**, **−9.56)**	−0.01 (−0.06, 0.04)	−7.37 (−51.51, 36.78)	
Q5	−56.01 (−121.80, 9.78)	−0.02 (−0.08, 0.03)	**−61.90 (−106.14**, **−17.67)**	
*P* for trend	**0.01**	0.29	**0.004**	
FT3 continuous	28.59 (−27.00, 84.18)	27.00 (−16.83, 70.83)	−5.00 (−42.63, 32.63)	0.16
FT3 quintiles				0.11
Q1	Reference	Reference	Reference	
Q2	−8.09 (−73.25, 57.07)	13.45 (−37.26, 64.16)	−32.40 (−75.83, 11.03)	
Q3	−22.96 (−89.76, 43.84)	31.77 (−17.19, 80.74)	−14.95 (−58.54, 28.64)	
Q4	21.63 (−46.58, 89.84)	22.05 (−27.75, 71.85)	−17.43 (−62.44, 27.59)	
Q5	18.48 (−45.75, 82.71)	37.27 (−14.08, 88.62)	−15.33 (−59.24, 28.58)	
*P* for trend	0.39	0.16	0.75	

a *Adjusted for maternal education, maternal height, maternal age, gestational age, parity, infant's sex, high risk pregnancy, and mode of delivery. Bold values are statistically significant*.

**Table 4:** Adjusted OR^a^ for low birth weight associated with maternal thyroid parameters during early pregnancy stratified by GWG among normal weight women (*N* = 6,010).

**Variables**	**GWG (kg)**			***P* for interaction**
	** <11.5 (*N* = 1,244) OR (95% CI)**	**11.5**~**16.0 (*N* = 2,001) OR (95% CI)**	**≥16.0 (*N* = 2,765) OR (95% CI)**	
TSH continuous	0.62 (0.35, 1.09)	1.43 (0.55, 2.45)	0.79 (0.60, 3.95)	0.16
TSH quintiles				0.37
Q1	Reference	Reference	Reference	
Q2	0.50 (0.18, 1.42)	1.01 (0.31, 3.28)	0.49 (0.15, 2.47)	
Q3	0.65 (0.24, 1.78)	1.03 (0.34, 3.10)	0.52 (0.12, 2.17)	
Q4	0.53 (0.16, 1.70)	0.85 (0.25, 2.91)	1.01 (0.29, 3.52)	
Q5	0.36 (0.10, 1.10)	1.55 (0.52, 4.56)	0.43 (0.10, 1.75)	
*P* for trend	0.10	0.49	0.46	
FT4 continuous	16.81 (0.42, 673.05)	4.22 (0.10, 182.13)	30.12 (0.33, 2791.18)	0.57
FT4 quintiles				0.69
Q1	Reference	Reference	Reference	
Q2	0.73 (0.22, 2.45)	1.13 (0.39, 3.25)	1.56 (0.28, 8.66)	
Q3	0.74 (0.21, 2.59)	0.90 (0.28, 2.84)	2.98 (0.58, 15.27)	
Q4	1.64 (0.54, 4.97)	1.37 (0.44, 4.26)	1.79 (0.34, 9.45)	
Q5	1.25 (0.41, 3.80)	1.60 (0.52, 4.90)	3.17 (0.67, 14.93)	
*P* for trend	0.30	0.38	0.15	
FT3 continuous	**2.52 (1.00, 6.36)**	1.80 (0.73, 4.48)	1.89 (0.68, 5.26)	0.93
FT3 quintiles				0.45
Q1	Reference	Reference	Reference	
Q2	1.43 (0.38, 5.41)	1.06 (0.29, 3.85)	0.88 (0.19, 4.01)	
Q3	**3.47 (1.08, 11.13)**	1.84 (0.58, 5.87)	1.04 (0.22, 4.88)	
Q4	1.34 (0.34, 5.23)	2.34 (0.75, 7.34)	1.22 (0.25, 5.98)	
Q5	3.17 (0.98, 10.22)	2.27 (0.70, 7.42)	2.25 (0.58, 8.73)	
*P* for trend	0.08	0.07	0.14	

a *Adjusted for maternal education, maternal height, maternal age, gestational age, parity, infant's sex, high risk pregnancy, and mode of delivery. Bold values are statistically significant*.

Among underweight and overweight women, inverse associations between maternal FT4 concentrations and birth weight were only observed in excessive GWG but not in inadequate and adequate GWG (Tables 5, 6); specially, the interaction between FT4 and GWG among overweight women was significant (Table 6). The birth weight decreased by 359.33 g (95% CI: −700.95, −17.72) and by 527.56 g (95% CI, −1007.85, −47.26) for every unit increase in FT4 concentrations in underweight and overweight women with excessive GWG, respectively. Similarly, the associations still persist when maternal FT4 concentrations were modeled as quintiles in overweight women. We did not observe any significant associations between maternal thyroid hormones and birth length within strata of GWG across pre-pregnancy BMI categories (Tables S2–S4).

**Table 5:** Adjusted regression coefficients ^a^ for birth weight associated with maternal thyroid parameters during early pregnancy stratified by GWG among underweight women (*N* = 1,358).

**Variables**	**GWG (kg)**			***P* for interaction**
	** <12.5 (*N* = 218) β (95% CI)**	**12.5**~**18.0 (*N* = 629) β (95% CI)**	**≥18.0 (*N* = 511) β (95% CI)**	
TSH continuous	−28.88 (−88.42, 30.69)	−32.32 (−69.84, 5.18)	8.98 (−36.66, 54.62)	0.14
TSH quintiles				0.12
Q1	Reference	Reference	Reference	
Q2	−84.88 (−214.02, 44.26)	−62.25 (−147.30, 22.80)	13.23 (−87.12, 113.58)	
Q3	−59.22 (−197.25, 78.82)	−31.96 (−112.30, 48.39)	4.08 (−96.42, 104.59)	
Q4	−92.62 (−232.62, 47.38)	**−95.04 (−179.42**, **−10.66)**	5.52 (−95.98, 107.02)	
Q5	−81.50 (−220.24, 57.23)	−63.96 (−147.76, 20.03)	16.75 (−83.51, 117.00)	
*P* for trend	0.29	0.09	0.82	
FT4 continuous	−223.01 (−666.41, 220.39)	−128.40 (−426.45, 169.66)	**−359.33 (−700.95**, **−17.72)**	0.14
FT4 quintiles				0.14
Q1	Reference	Reference	Reference	
Q2	53.41 (−84.30, 191.11)	15.60 (−79.82, 111.02)	0.86 (−106.23, 107.94)	
Q3	20.64 (−113.42, 154.69)	−63.94 (−160.69, 32.82)	16.15 (−94.28, 126.57)	
Q4	−50.88 (−191.75, 89.98)	−7.56 (−101.78, 86.66)	−27.59 (−135.27, 80.09)	
Q5	−58.94 (−191.20, 89.98)	−18.90 (−112.74, 74.94)	−96.60 (−202.68, 9.49)	
*P* for trend	0.15	0.58	**0.04**	
FT3 continuous	29.50 (−87.30, 146.29)	−12.07 (−87.87, 63.74)	15.31 (−79.22, 109.84)	0.80
FT3 quintiles				0.26
Q1	Reference	Reference	Reference	
Q2	34.63 (−86.28, 155.54)	−27.16 (−107.67, 53.36)	−30.87 (−127.31, 65.56)	
Q3	19.83 (−105.27, 144.93)	20.43 (−59.71, 100.57)	29.85 (−69.69, 129.40)	
Q4	60.87 (−74.26, 196.00)	−12.54 (−98.26, 73.19)	−8.14 (−113.60, 97.32)	
Q5	−4.56 (−157.30, 148.18)	−29.97 (−117.21, 57.27)	37.83 (−71.34, 146.99)	
*P* for trend	0.31	0.69	0.40	

a *Adjusted for maternal education, maternal height, maternal age, gestational age, parity, infant's sex, high risk pregnancy, and mode of delivery. Bold values are statistically significant*.

**Table 6:** Adjusted regression coefficients^a^ for birth weight associated with maternal thyroid parameters during early pregnancy stratified by GWG among overweight women (*N* = 625).

**Variables**	**GWG (kg)**			***P* for interaction**
	** <7.0 (*N* = 129) β (95% CI)**	**7.0**~**11.5 (*N* = 131) β (95% CI)**	**≥11.5 (*N* = 365) β (95% CI)**	
TSH continuous	41.32 (−71.78, 154.42)	−7.02 (−99.64, 85.60)	−21.74 (−84.11, 40.63)	0.49
TSH quintiles				0.56
Q1	Reference	Reference	Reference	
Q2	29.03 (−208.69, 266.76)	−22.07 (−232.12, 187.98)	8.26 (−133.29, 149.80)	
Q3	27.58 (−205.26, 266.41)	81.67 (−151.27, 314.62)	82.83 (−67.36, 233.02)	
Q4	71.37 (−159.36, 302.10)	62.38 (−163.27, 288.04)	−79.13 (−217.46, 59.20)	
Q5	42.12 (−207.322, 29.56)	−55.90 (−287.27, 175.46)	3.65 (−136.78, 144.08)	
*P* for trend	0.62	0.99	0.56	
FT4 continuous	−620.52 (−1450.11, 209.06)	−237.90 (−967.99, 492.19)	**−527.56 (−1007.85**, **−47.26)**	**0.03**
FT4 quintiles				**0.01**
Q1	Reference	Reference	Reference	
Q2	−179.60 (−384.01, 24.81)	−30.95 (−217.81, 155.92)	**−133.14 (−249.37**, **−16.91)**	
Q3	−169.96 (−380.01, 24.81)	−15.69 (−235.99, 204.61)	**−162.25 (−299.42**, **−25.09)**	
Q4	−254.99 (−510.05, 0.06)	−21.83 (−226.77, 183.11)	**−191.98 (−325.74**, **−58.22)**	
Q5	−125.78 (−391.42, 139.85)	−114.88 (−342.91, 113.14)	−130.71 (−284.93, 23.51)	
*P* for trend	0.14	0.43	**0.01**	
FT3 continuous	27.63 (194.82, 250.09)	94.03 (−109.46, 297.52)	−18.72 (−139.92, 102.48)	0.76
FT3 quintiles				0.59
Q1	Reference	Reference	Reference	
Q2	15.93 (−280.56, 312.42)	0.14 (−197.78, 479.29)	−101.65 (−250.70, 47.40)	
Q3	65.46 (−192.62, 323.53)	0.05 (−261.75, 353.88)	−135.76 (−282.70, 11.18)	
Q4	118.411 (−144.77, 381.60)	125.06 (−182.35, 0.43)	22.98 (121.93, 167.88)	
Q5	37.491 (−216.29, 291.27)	123.10 (−170.31, 416.51)	−111.56 (−252.38, 29.26)	
*P* for trend	0.66	0.50	0.59	

a *Adjusted for maternal education, maternal height, maternal age, gestational age, parity, infant's sex, high risk pregnancy, and mode of delivery. Bold values are statistically significant*.

## Discussion

In this large prospective study of Chinese population, we investigated the effects of maternal thyroid parameters including TSH, FT3, and FT4 within the normal ranges during early pregnancy on birth outcomes. We observed that maternal TSH and FT4 concentrations were negatively associated with birth weight and the latter only occurred in normal weigh women with inadequate and excessive GWG, as well as in both underweight and overweight women with excessive GWG. We also found that maternal FT4 and FT3 concentrations were associated with increased risk for low birth weight and the latter only occurred in normal weigh women with inadequate GWG. Our results suggest that maternal normal thyroid function during early pregnancy influences fetal growth and GWG modifies the effects.

Maternal thyroid hormones are required for normal development and growth of the developing fetus. Accumulating studies have shown that maternal thyroid dysfunction such as overt hypothyroidism and hyperthyroidism during pregnancy is associated with preterm birth, low birth weight, and fetal death (11, 12, 27, 28). Several meta-analyses have also concluded that maternal subclinical hypothyroidism is related with increased risk of preterm birth and intrauterine growth restriction (16, 29). However, only limited studies have examined the effects of maternal normal thyroid function during pregnancy on birth outcomes. Medici et al. found that mothers with normal-range FT4 concentrations during early pregnancy were related with reduced birth weight and increased risk of low birth weight (19). Shield et al. also observed an inverse association between maternal FT4 concentrations at 28 weeks of gestation and birth weight among normal and healthy mothers (20). In a Chinese population, Zhang et al. found that maternal high-normal FT4 concentrations (between 90th and 97.5th percentiles) during early and late pregnancy were associated with reduced birth weight (22). Our results of maternal FT4 concentrations within the normal range during early pregnancy in relation to reduced birth weight and increased risk of low birth weight were consistent with these previous studies. Moreover, we firstly found that mothers with normal-range FT4 concentrations during early pregnancy were also negatively related with birth length. These findings suggest that alternation in maternal FT4 concentrations within the normal range during early pregnancy can influence fetal growth. A potential mechanism underlying the observed findings is the placental transfer of high-normal maternal FT4 to the fetus, which influences the fetal hypothalamus-pituitary-thyroid axis function during early pregnancy (19, 22). Another potential mechanism is that high-normal maternal FT4 increases the degradation of proteins and lipids, which results in a lacking state of maternal chronic caloric and further adversely affects fetal growth (30, 31).

In the present study, maternal normal-range TSH concentrations during early pregnancy were also observed to be related with reduced birth weight. In line with our result, Zhang et al. also observed that maternal higher TSH concentrations during early and late pregnancy were associated with lower birth weight (22). In a prospective cohort study in Crete, Greece, Karakosta et al. reported that higher TSH concentrations within the reference range during early pregnancy were associated with increased risk of low birth weight (8). However, Medici et al. reported no significant association between maternal normal-range TSH concentrations during early pregnancy and birth weight (19). Differences in population characteristics and sample sizes, as well as varying adjusted confounders in analytical models may partly contribute to the discrepancy between studies.

FT3, as the principal bioactive thyroid hormone, can act directly through anabolic effects on fetal metabolism and the stimulation of fetal oxygen consumption (1). Few studies to date have investigated the associations between maternal FT3 concentrations and birth outcomes. In this study, we firstly found that maternal normal-range FT3 concentrations during early pregnancy were associated with increased risk for low birth weight. However, a previous study showed a positive association between maternal FT3 concentrations throughout pregnancy and birth weight (22). The mechanism that maternal FT3 affects fetal growth is not clear. More population-based prospective studies and mechanistic investigations should be conducted into this topic.

GWG reflects various characteristics including maternal fluid expansion, fat accumulation, placenta and the growth of the fetus (32) and appropriate GWG is necessary to ensure normal fetal growth (23). Studies have shown that excessive and insufficient GWG are related with adverse birth outcomes such as low birth weight, preterm birth, small for gestational age and macrosomia (23, 33). In the present study, we firstly examined the effect modification by GWG on the associations between maternal normal thyroid function during early pregnancy on birth outcomes. After stratification by pre-pregnancy BMI category and GWG, we found that maternal FT4 concentrations in relation to decreased birth weight occurred in normal weigh women with inadequate and excessive GWG, as well as in underweight and overweight women with excessive GWG and maternal FT3 concentrations in relation to increased risk for low birth weight occurred in normal weigh women with inadequate GWG. The potential mechanisms underlying this are not fully understood. Excessive or inadequate GWG may alter the hormone levels of leptin, which affects the thyrotropic axis and the secretion of thyroid hormones and thus influences the fetal growth (34). Pregnant women with excessive GWG have been shown to have higher TSH and lower FT4 concentrations compared with those with appropriate GWG (35).

The large sample size allowing us to examine the effect modification by GWG across pre-pregnancy BMI categories was the main strength of this study. In addition, simultaneous measurements of TSH, FT3, and FT4 concentrations for the comprehensive assessment of thyroid function were another strength of this study. However, several limitations in our study should be noted. First, we only measured thyroid hormone levels in the first trimester of pregnancy. Some studies have shown that maternal thyroid function during late pregnancy is associated with adverse birth outcomes (20, 22). Therefore, future studies should further identify the critical windows for the effects of maternal thyroid hormones during pregnancy on birth outcomes. Second, we did not assess maternal iodine status and thyroid peroxidase antibody, which has been associated with thyroid function and/or fetal growth (9, 36). Finally, the recruitment was restricted to a single hospital and thus the generalizability of our results to a wider population should be cautious.

In summary, in this cohort of Chinese pregnant women at early pregnancy, we found that alternations in maternal TSH, FT4, and FT3 concentrations within the normal ranges can influence fetal growth. Specially, maternal FT4 concentrations in relation to decreased birth weight occurred in normal weigh women with inadequate and excessive GWG, as well as in underweight and overweight women with excessive GWG. Moreover, maternal FT3 concentrations in relation to increased risk for low birth weight occurred in normal weigh women with inadequate GWG. Our results suggest that maternal normal thyroid function during early pregnancy with excessive and inadequate GWG may adversely influence fetal growth.

## Data Availability Statement

All datasets generated for this study are included in the article/Supplementary Material.

## Ethics Statement

The studies involving human participants were reviewed and approved by Medical Ethics Committee of Wuhan Children's Hospital (Wuhan Maternal and Child Healthcare Hospital). Written informed consent to participate in this study was provided by the participants and the participants' legal guardian/next of kin.

## Author Contributions

X-JH and BZ contributed to study design, writing, and revision of the manuscript. YC and LL contributed to the assays. W-QC and BZ performed data analysis. All authors contributed to the article and approved the submitted version.

## Conflict of Interest

YC was employed Wuhan Pengxiang Medical Equipment Co., Ltd. The remaining authors declare that the research was conducted in the absence of any commercial or financial relations that could be construed as a potential conflict of interest.
