# Supplementary material for: Effect of Gestational Weight Gain on Associations Between Maternal Thyroid Hormones and Birth Outcomes
Source: Front Endocrinol (Lausanne). 2020 Sep 3;11:610. doi: 10.3389/fendo.2020.00610 (PMC7494749; doi:10.3389/fendo.2020.00610)
Supplement: Supplementary file 1 [file Data_Sheet_1.PDF]

Table S1. Recommendations for GWG during pregnancy.

Recommendation	Pre-pregnancy weight			
	Underweight	Normal weight	Overweight	Obese
Pre-pregnancy BMI	<18.5	18.5~24.9	25.0~29.9	≥ 30
Total weight gain range, kg	12.5~18.0	11.5~16.0	7~11.5	5.0~9.0

Table S2. Adjusted regression coefficients [a] for birth length associated with maternal thyroid parameters during early pregnancy stratified by GWG among underweight women (N=1358).

Variables	GWG (kg)			*P* for interaction
	<12.5 (N=218) β (95%CI)	12.5~18.0 (N=629) β (95%CI)	≥18.0 (N=511) β (95%CI)	
TSH continuous	-0.01 (-0.26, 0.24)	-0.08 (-0.23, 0.07)	0.10 (-0.08, 0.28)	0.20
TSH quintiles				0.15
Q1	Reference	Reference	Reference	
Q2	-0.27 (-0.82, 0.27)	-0.15 (-0.49, 0.20)	-0.19 (-0.58, 0.19)	
Q3	-0.02 (-0.60, 0.57)	0.03 (-0.29, 0.36)	-0.07 (-0.46, 0.31)	
Q4	-0.32 (-0.91, 0.27)	-0.13 (-0.47, 0.21)	0.06 (-0.33, 0.45)	
Q5	-0.16 (-0.74, 0.42)	-0.18 (-0.51, 0.16)	0.08 (-0.31, 0.46)	
P for trend	0.62	0.38	0.38	
Free T4 continuous	-1.53 (-3.38, 0.33)	-0.62 (-1.82, 0.58)	-0.60 (-1.91, 0.72)	0.14
Free T4 quintiles				0.72
Q1	Reference	Reference	Reference	
Q2	-0.07(-0.64, 0.51)	-0.007 (-0.39, 0.38)	0.19 (-0.225, 0.601)	
Q3	0.070 (-0.49, 0.63)	-0.24 (-0.63, 0.15)	0.19 (-0.238, 0.614)	
Q4	-0.305 (-0.89, 0.28)	-0.13 (-0.51, 0.25)	0.17 (-0.250, 0.581)	
Q5	-0.522 (-1.07, 0.03)	-0.13 (-0.51, 0.24)	-0.11 (-0.52, 0.30)	
P for trend	0.041	0.383	0.452	
Free T3 continuous	-0.11 (-0.60, 0.38)	-0.13 (-0.43, 0.18)	0.043 (-0.321, 0.406)	**0.04**
Free T3 quintiles				0.09
Q1	Reference	Reference	Reference	
Q2	0.28 (-0.22, 0.79)	-0.07 (-0.39, 0.26)	0.29 (-0.08, 0.66)	
Q3	0.13 (-0.39, 0.65)	0.05 (-0.27, 0.37)	0.30 (-0.08, 0.69)	
Q4	0.15 (-0.42, 0.72)	0.03 (-0.31, 0.38)	-0.005 (-0.41, 0.40)	
Q5	-0.16 (-0.80, 0.48)	-0.27 (-0.62, 0.08)	0.27 (-0.15, 0.69)	
P for trend	0.74	0.32	0.64	

[a]Adjusted for maternal education, maternal height, maternal age, gestational age, parity, infant's sex, high risk pregnancy scores, and mode of delivery.

Table S3. Adjusted regression coefficients [a] for birth length associated with maternal thyroid parameters during early pregnancy stratified by GWG among normal weight women (N=6010).

Variables	GWG (kg)			*P* for interaction
	<11.5 (N=1244) β (95%CI)	11.5~16.0 (N=2001) β (95%CI)	≥16.0 (N=2765) β (95%CI)	
TSH continuous	-0.02 (-0.14, 0.09)	-0.05 (-0.16, 0.06)	-0.03 (-0.09, 0.04)	0.63
TSH quintiles				1.00
Q1	Reference	Reference	Reference	
Q2	-0.06 (-0.31, 0.20)	0.03 (-0.03, 0.38)	-0.05 (-0.20, 0.11)	
Q3	-0.13 (-0.39, 0.13)	-0.21 (-0.46, 0.04)	-0.06 (-0.21, 0.10)	
Q4	0.08 (-0.26, 0.27)	0.03 (-0.22, 0.28)	-0.06 (-0.22, 0.09)	
Q5	-0.07 (-0.34, 0.19)	-0.07 (-0.32, 0.18)	-0.07 (-0.22, 0.08)	
P for trend	0.763	0.604	0.347	
Free T4 continuous	-0.77 (-1.64, 0.11)	-0.52 (-1.36, 0.33)	-0.30 (-0.81, 0.21)	0.49
Free T4 quintiles				0.62
Q1	Reference	Reference	Reference	
Q2	0.06 (-0.21, 0.32)	-0.06 (-0.30, 0.19)	0.05 (-0.10, 0.20)	
Q3	-0.01 (-0.27, 0.26)	-0.10 (-0.35, 0.14)	0.08 (-0.07, 0.23)	
Q4	-0.14 (-0.41, 0.13)	-0.11 (-0.36, 0.14)	0.01 (-0.15, 0.16)	
Q5	-0.16 (-0.42, 0.11)	-0.10 (-0.35, 0.15)	-0.07 (-0.22, 0.08)	
P for trend	0.10	0.37	0.31	
Free T3 continuous	0.04 (-0.18, 0.26)	0.09 (-0.13, 0.30)	-0.01 (-0.14, 0.12)	0.43
Free T3 quintiles				0.44
Q1	Reference	Reference	Reference	
Q2	0.03 (-0.23, 0.29)	0.12 (-0.13, 0.37)	-0.09 (-0.24, 0.06)	
Q3	-0.08 (-0.34, 0.19)	0.13 (-0.12, 0.37)	-0.05 (-0.20, 0.10)	
Q4	0.01 (-0.26, 0.29)	0.07 (-0.18, 0.32)	-0.03 (-0.18, 0.13)	
Q5	0.01 (-0.24, 0.27)	0.14 (-0.12, 0.40)	-0.03 (-0.18, 0.12)	
P for trend	0.98	0.43	0.96	

[a]Adjusted for maternal education, maternal height, maternal age, gestational age, parity, infant's sex, high risk pregnancy scores, and mode of delivery.

Table S4. Adjusted regression coefficients [a] for birth length associated with maternal thyroid parameters during early pregnancy stratified by GWG among overweight women (N=625).

Variables	GWG (kg)			*P* for interaction
	<7.0 (N=129) β (95%CI)	7.0~11.5 (N=131) β (95%CI)	≥11.5 (N=365) β (95%CI)	
TSH continuous	-0.12 (-0.47, 0.23)	0.03(-0.32, 0.38)	-0.03 (-0.23, 0.18)	0.81
TSH quintiles				0.85
Q1	Reference	Reference	Reference	
Q2	0.28 (-0.45, 1.01)	-0.24 (-1.02, 0.55)	0.24(-0.22, 0.71)	
Q3	-0.07 (-0.78, 0.65)	0.40 (-0.46, 1.27)	0.26(-0.23, 0.76)	
Q4	0.18(-0.53, 0.89)	0.18 (-0.66, 1.02)	-0.15(-0.60, 0.31)	
Q5	-0.28 (-1.04, 0.49)	-0.11 (-0.97, 0.76)	0.18 (-0.29, 0.64)	
P for trend	0.52	0.71	0.85	
Free T4 continuous	-2.15 (-4.17, 0.41)	-2.35 (-5.05, 0.35)	-1.08 (-2.66, 0.51)	0.18
Free T4 quintiles				
Q1	Reference	Reference	Reference	
Q2	-0.21 (-0.85, 0.43)	-0.27 (-0.96, 0.42)	-0.23 (-0.61, 0.16)	
Q3	-0.13 (-0.78, 0.53)	-0.13 (-0.94, 0.69)	-0.32 (-0.78, 0.13)	
Q4	-0.47 (-1.27, 0.33)	-0.52 (-1.28, 0.24)	-0.44 (-0.88, 0.01)	
Q5	-0.68 (-1.51, 0.15)	-0.73 (-1.57, 0.11)	-0.30 (-0.81, 0.21)	
P for trend	0.10	0.07	0.07	
Free T3 continuous	-0.26 (-0.95, 0.43)	0.14 (-0.62, 0.91)	0.07 (-0.33, 0.47)	0.72
Free T3 quintiles				
Q1	Reference	Reference	Reference	
Q2	-0.27 (-1.18, 0.65)	0.24 (-1.03, 1.51)	-0.10 (-0.59, 0.39)	
Q3	-0.14 (-0.94, 0.66)	0.12 (-1.04, 1.27)	-0.22 (-0.70, 0.27)	
Q4	0.02 (-0.80, 0.83)	0.17 (-0.98, 1.33)	0.29 (-0.19, 0.77)	
Q5	-0.27 (-1.05, 0.52)	0.20 (-0.91, 1.30)	-0.12 (-0.58, 0.35)	
P for trend	0.70	0.83	0.76	

[a]Adjusted for maternal education, maternal height, maternal age, gestational age, parity, infant's sex, high risk pregnancy scores, and mode of delivery.
